# Thwarted belongingness, perceived burdensomeness, and acquired capability for suicide among university students of Bangladesh: Scales validation and status assessment

**DOI:** 10.3389/fpsyt.2022.1025976

**Published:** 2022-10-14

**Authors:** S. M. Yasir Arafat, Fahad Hussain, Mir Susmita Zaman, Tamanna Tabassum, Md. Khayrul Islam, Farzana Rabin Shormi, Anisur Rahman Khan, Md. Rabiul Islam, A. S. M. Redwan, Noor Ahmed Giasuddin, Anila Mubashir, Md. Abdullah Saeed Khan

**Affiliations:** ^1^Department of Psychiatry, Enam Medical College and Hospital, Dhaka, Bangladesh; ^2^Department of Pharmacy, Noakhali Science and Technology University, Noakhali, Bangladesh; ^3^Pi Research Consultancy Center, Dhaka, Bangladesh; ^4^Department of Psychiatry, Tairunnessa Memorial Medical College, Gazipur, Bangladesh; ^5^Department of Psychiatry, Holy Family Red Crescent Medical College, Dhaka, Bangladesh; ^6^Faculty of Liberal Arts and Social Sciences, East West University, Dhaka, Bangladesh; ^7^Department of Pharmacy, University of Asia Pacific, Dhaka, Bangladesh; ^8^Department of Psychiatry, Chattogram International Medical College, Chattogram, Bangladesh; ^9^Department of Psychiatry, Shaheed Tajuddin Ahmad Medical College, Gazipur, Bangladesh; ^10^Department of Applied Psychology, National University of Modern Languages, Rawalpindi, Pakistan

**Keywords:** suicide in Bangladesh, interpersonal needs theory, students, psychometric properties, validity, reliability

## Abstract

**Background:**

Identification of prior mental events of suicide attempts has immense importance in suicide prevention. However, it has not been studied in Bangladesh as there was no available psychometrically valid instrument measuring it.

**Objectives:**

We aimed to test the psychometric properties of the interpersonal needs questionnaire (INQ-15) and acquired capability for suicide scale-fearlessness about death (ACSS-FAD) in Bangla along with the determination of the level of thwarted belongingness, perceived burdensomeness, and acquired capability for suicide.

**Materials and methods:**

We collected data between 29 March and 14 April 2022 from 1,207 students of medical colleges and universities in Bangladesh by *Google form*. We assessed the psychometric properties of Bangla INQ and ACSS-FAD scales and examined factors associated with thwarted belongingness, perceived burdensomeness, and acquired capability for suicide.

**Results:**

The mean age of the participants was 22.82 ± 1.68 (range 18–29) years, 51% were females, 84% were graduate students, and 92% were unmarried. Both of the scales revealed acceptable levels of reliability. Confirmatory factor analysis revealed a two-factor structure of Bangla INQ after dropping three items from thwarted belongingness domain (item 9, 11, and 12) and a single factor structure for Bangla ACSS-FAD after dropping three items (item 1, 4, and 6). Perceived burdensomeness was significantly higher in females, students with a history of mental illness, family history of suicide, and the history of suicidal attempts. Fearlessness about death was significantly higher among females, non-Muslim participants, and history of suicidal attempts.

**Conclusion:**

The current study revealed psychometric properties of two suicide scales (INQ and ACSS-FAD) in Bangla that can be used in subsequent studies. Prevention strategies targeting to females, persons with psychiatric disorder, history of previous attempt(s) should be prioritized specially among the young age group.

## Introduction

Death by suicide is a global social and public health concern. It is a complicated phenomenon that brings suffering to hundreds of thousands of people around the world. The World Health Organization (WHO) estimates that the global suicide rate in 2019 was 9.0 per 100,000 people; with 77% of these suicides occurring in low- and middle-income countries (LMICs) (1). It is the fourth leading cause of death between 15 and 19 years and 88% of adolescent suicides happen in LMICs (1). Suicide is a multi-factorial act that develops over time through a variety of life experiences (2). It comprises a series of events that begins with suicidal thoughts, plans, and progresses through increasingly fatal acts. It is influenced by a range of proximal and distal risk factors, including psychopathology, particularly major depressive illness and psychoactive substance abuse, as well as life events, family background, and personality constructs (3, 4).

The *Interpersonal–Psychological Theory of Suicide (IPTS)* is one of the few empirically tested theoretical models explaining why people die by suicide (5, 6). According to the IPTS, attempting suicide requires both the desire and the ability to carry out the act. Hence, people are most at risk for suicide when they have three distinct conditions: (a) they comprehend themselves to be a burden to others i.e., perceived burdensomeness (PB); (b) they have a profound sense of disconnectedness from others i.e., thwarted belongingness (TB); and (c) they have habituated to the fear and physical pain inherent in a suicidal act by repeatedly enduring painful and provocative situations (7). The simultaneous existence of high levels of all three components is a theorized prelude to increased levels of suicide risk.

The *Interpersonal Needs Questionnaire* (INQ) was developed to examine thwarted belongingness and perceived burdensomeness, by Van Orden (7, 8). Several version of INQ has been adapted from the original INQ-25. Among them, the INQ-15 has a strong internal consistency and supports as a two-factor model (representing the PB and TB structures) (9). The acquired capability for suicide (ACS) is the theorized cognitive capability allowing conversion of suicidal thought to behavior. Reduced fear of death and increased pain tolerance are two components of acquired competence that emerges over time as a result of repeated exposure to physically uncomfortable and/or fear-inducing experiences (i.e., painful and provocative events) (10). The hypothesis proposes that systems underlying fearlessness about death and dying are important for forming suicidal intent whilst the increased pain tolerance is required for attempting a lethal or near-lethal suicide. The Acquired Capability for Suicide Scale (ACSS) was created to address the requirement. It is a 20-item self-report scale that assesses levels of fearlessness about death, pain tolerance, and painful and provocative incidents (11, 12). Many countries have psychometrically validated two iterations of the original ACSS, a 5-item scale and a 7-item sub-scale that only evaluates fearlessness about death (ACSS-FAD) (13, 14).

Suicide is an under-researched topic in the LMICs where Bangladesh is not an exception. There are scarce data assessing the prior events of suicide attempts resulting in difficulties in ascertaining the risk factors and formulating the prevention strategies. Available evidence indicates that the majority of the suicides happens before the 30 years of age (15). One recent study identified that 23.8% of under-graduate medical students had life-time suicidal ideation in Bangladesh (16). One study from 12 countries identified the prevalence of suicidal ideation is approximately 29% of university (17). Suicidal ideation may indicate mental health problem, risky sexual and criminal behaviors which consume health services (18). One study identified that suicidal attempt is an important predictor for readmission among youths with severe mental illness (19). Psychometrically valid scales in native languages would help to identify the mental events of suicidal behavior, promote research and better mental health services as well (20, 21). However, no previous attempt has been identified to validate such instruments in Bangla. Therefore, this study is undertaken to perform psychometric validation of two scales (INQ-15 and ACSS-FAD-7) of suicide in Bangla. The findings of this study would be considered baseline indicators in Bangladesh and would foster further studies in the country.

## Materials and methods

### Study place and procedure

We conducted a cross-sectional study where responses were collected in *Google form*. Data were collected conveniently from students of five purposively selected medical colleges (private 4, public 1) and five purposively selected universities (private 2, public 3) between March 29 and 14 April 2022. Here is the name of the institutions-Chattagram International Medical College, Enam Medical College, Holy Family Red Crescent Medical College, Shaheed Tajuddin Ahmed Medical College, Tairunnessa Memorial Medical College, East West University, Mawlana Bhashani Science and Technology University, Noakhali Science and Technology University, University of Asia Pacific, and University of Liberal Arts Bangladesh. We sent the response link through the class representatives and requested to distribute it among all the students. The questionnaire contained an introductory page mentioning the objectives of the study, a brief study procedure, details of the principal investigator as a point of query, the process of keeping anonymity, and rights to withdraw. Subsequently, informed electronic consent was sought. After confirming the consent the rest parts appeared. We considered adult Bangla-speaking Bangladeshi students as the inclusion criteria. Students from other countries were excluded from the study. Data were cleaned in Microsoft Excel software. Only the first and second authors have the access to the data who acted as data custodians for this study. We collected the email addresses of the participants to check the duplicate responses and we did not find any duplicate responses in the study.

### Instruments

#### Sociodemographic variables

We used the sociodemographic part of our previously conducted study among students (22). We considered age, sex, nuptiality, academic institute, faculty, current enrollment year, religious belief, family structure (nuclear/joint), monthly family income in taka (BDT), any history of chronic disease, psychiatric illness, drug history, and family history of suicidal behavior.

#### Suicidality

We included items in this section from our previously conducted study among students (22). We collected data on suicidal ideas/thoughts in the past year and lifetime, suicide plans, previous non-fatal-attempt, and sharing reporting of the suicidal idea. Did you have any idea of killing yourself in your lifetime? Did you have any idea of killing yourself in the past year? Have you ever made any plan to kill yourself? Did you have any attempt of suicide? Have you shared your suicidal thoughts to anyone?

#### Bangla interpersonal needs questionnaire

We adopted the Bangla Interpersonal Needs Questionnaire (INQ-B) from the original instrument developed by Van Orden et al. in 2012 (20). It consists of 15 items in two domains. The first six items comprised the perceived burdensomeness domain and the last nine items assess thwarted belongingness. Among the 15 items- items 7, 8, 10, 13, 14, and 15 were reverse coded. Responses were collected on 7 point scale indicating 1 for *not at all true for me* and 7 for *very true for me* with a neutral value of 3 for *somewhat true for me* (20).

#### Bangla acquired capability for suicide scale-fearlessness about death

We adopted the Bangla Acquired Capability for Suicide Scale-Fearlessness About Death (ACSS-FAD-B) from the original instrument developed by Ribeiro et al. in 2014 (11). It consists of seven items with a one-dimensional structure. Among the seven items- items 2, 3, and 5 were reverse coded. Responses were collected on 5 point scale indicating 0 for *not at all like me* and 4 for *very much like me* (11).

### Adaptation of INQ-15 and ACSS-FAD into Bangla

We adapted the instruments by following recommended guidelines of forward-backward translation (23). We performed two forward translations (performed by medical graduate-disguised and general person-undisguised). We compared and contrasted those two forward translations to compile into one version after addressing linguistic discrepancies. Then we performed two back-translations (performed by medical graduate-undisguised and lay person-disguised) of the compiled forward version into English. All the translators use Bangla as mother tongue and fluent in English. Likewise, the forward translations back-translations were compared, contrasted and compiled. Subsequently, an expert committee was formed for the validation study where all the translations were produced to compile the instrument for pretest. We performed pretest among 23 lay persons to check the comprehensibility and made final adjustment. No item was added, deleted, modified during this adaptation process.

### Data analysis

We analyzed data by IBM SPSS version 28.0 software, and Stata version 16. Confirmatory factor analysis was carried out through IBM SPSS AMOS (analysis of moment structure) version 25.0 using structural equation modeling (SEM). We mentioned the frequency and percentages of variables related to socio-demography and suicidality. We assessed the psychometric properties of INQ-B and ACSS-FAD-B on the basis of standard guidelines (24). We measured the reliability in the form of internal consistency expressed as Cronbach’s alpha coefficient where a cut-off of ≥0.70 was considered acceptable. We assessed the face and content validities during the translation and back-translation stages (19). Construct validity was assessed by performing the factor analysis. We performed both exploratory and confirmatory factor analysis to assess the construct validity of INQ-B and ACSS-FAD-B. We conducted *Principal Component Analysis* with *varimax rotation*, and visualized the scree plot. The normality of the INQ-Bangla score and the ACSS-FAD score was checked using the Shapiro-Wilk test, histogram with Gaussian curve, and Q-Q plot. As the data followed a skewed distribution, comparisons of these scores across variables with binary categories were conducted using the Mann-Whitney *U* test. Data were presented as median (Interquartile Range).

### Ethical aspects

Formal permission was obtained from the copyright holder Professor Thomas Joiner on 14 January 2022, before initiating the project. The study was approved by the ethical review committee of Enam Medical College on 07 March 2022 (EMC/ERC/2022/03-2). We ensured informed consent electronically from the students prior to starting the survey. Strict anonymity was ensured and only the first author acted as data gatekeeper.

## Results

We received 1,219 responses. Among them, seven participants did not have consent and an additional five had incomplete responses. Therefore, 12 responses were excluded from the analysis, and finally, we analyzed 1,207 responses. The mean age of the participants was 22.82 ± 1.68 years (range: 18–29 years). Among the 1,207 students, 615 (50.95%) were females, 1,017 (84.26%) were graduate students, 1,113 (92.21%) were never-married, 1,054 (87.32%) were Muslim and 1,006 (83.34%) belonged to nuclear families (Table 1). Among the participants, 720 (59.65%) were studying in universities and the rest 40.35% were studying in medical colleges, 140 (11.59%) had a history of mental illness, 142 (11.76%) had a family history of suicidal attempts, 66 (5.46%) had a family history of suicide, and 154 (12.75%) had past suicidal attempts (Table 1).

**TABLE 1:** Sociodemographic variables of participants (*n* = 1,207).

Variable	Category	n (%)
Sex	Male	591 (48.96)
	Female	615 (50.95)
	Others	1 (0.08)
Education	HSC (Grade 12)	1,017 (84.26)
	Graduate	190 (15.74)
Marital status	Unmarried	1,113 (92.21)
	Married	92 (7.62)
	Divorced	2 (0.16)
Religion	Islam	1,054 (87.32)
	Hindu	129 (10.69)
	Christian	6 (0.49)
	Buddhist	12 (0.99)
	Others	6 (0.49)
Study year	1st year	137 (11.35)
	2nd year	188 (15.57)
	3rd year	281 (23.28)
	4th year	331 (27.42)
	5th year	161 (13.33)
	Post graduate	109 (9.03)
Faculty	Medicine	487 (40.35)
	University	720 (59.65)
Family type	Nuclear	1,006 (83.34)
	Joint	201 (16.65)
Chronic illness	Yes	209 (17.31)
Taking medication	Yes	159 (13.17)
History of mental illness	Yes	140 (11.59)
Family history of suicide attempt	Yes	142 (11.76)
Family history of suicide	Yes	66 (5.46)
Lifetime suicidal thought	Yes	623 (51.61)
Last year suicidal thought	Yes	339 (28.08)
History of suicidal attempt	Yes	154 (12.75)
**Total**		**1,207 (100)**

We performed exploratory factor analysis with *Principal Component Analysis* with *varimax rotation* to assess the Kaiser-Meyer-Olkin (KMO) Measure of Sampling Adequacy, internal consistency form of reliability measured by Cronbach’s alpha, initial and rotated factor loadings. The Kaiser-Meyer-Olkin (KMO) Measure of Sampling Adequacy of INQ-B was 0.88 which was statistically significant (*p* = 0.0001) and ACSS-FAD-B was 0.73 which was also statistically significant (*p* = 0.0001). The internal consistency of the INQ-B was measured by Cronbach’s alpha which was 0.84 for the total scale, 0.92 for the PB subscale, and 0.87 for the TB subscale. The initial Cronbach’s alpha of TB subscale with nine items was 0.75. However, after dropping the three items (item 9, 11, and 12) based on factor analysis it revealed 0.87 with six items. The internal consistency of the ACSS-FAD-B was measured by Cronbach’s alpha which was 0.76 which is acceptable. The initial Cronbach’s alpha of ACSS-FAD-B with seven items was 0.65. However, after dropping the three items (item 2, 4, and 6) based on factor analysis it revealed 0.76 with four items. The dropped three items (item 2, 4, and 6) revealed a Cronbach’s alpha of 0.49. The two factors of INQ-B and one factor of ACSS-FAD-B cover 66.02% (37.63 and 28.40%) and 60.34% of the variance respectively.

**FIGURE 1:**
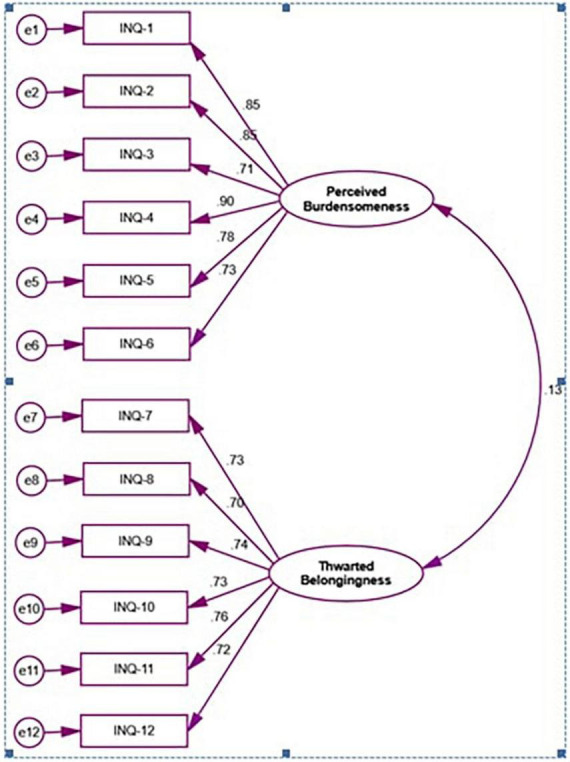
First order confirmatory factor analysis of INQ-B.

The mean score of responses of the items in INQ-B is presented in Table 2. The factor rotation revealed acceptable values (0.69-0.90) in PB scale. Among the nine items of TB domain, three items (item 9, 11, and 12) took poor loading (0.14, 0.04, and 0.01 respectively) (Table 2). These three items again showed poor inter-item correlation i.e., 0.13, 0.04, and 0.02 respectively (Table 2). We considered to drop items with a value of <0.30 (13, 19). Based on these factors these three items have been dropped from the final construct. Therefore, the INQ-B contains twelve items with six items in each domain of perceived burdensomeness and thwarted belongingness domain (Figure 1).

**TABLE 2:** Construct and factor loading of INQ-B (*n* = 1,207).

			Factor loading				
			
Item	Mean	Std. deviation	Perceived burdensomeness	Thwarted belongingness	Corrected item-total correlation	Inter-item correlation*	Cronbach’s alpha if item deleted
INQ-1	1.79	1.61	0.87		0.56	1	0.82
INQ-2	1.93	1.70	0.86		0.58	0.79	0.82
INQ-3	2.15	1.88	0.69		0.56	0.56	0.82
INQ-4	1.75	1.59	0.90		0.61	0.76	0.82
INQ-5	1.65	1.51	0.82		0.55	0.62	0.82
INQ-6	2.33	1.97	0.71		0.57	0.59	0.82
INQ-7r	4.36	2.23		0.77	0.44	1	0.82
INQ-8r	4.50	2.17		0.76	0.35	0.66	0.83
INQ-9*	2.93	2.08		**−0.14**	0.19	–0.13	0.84
INQ-10r	4.03	2.33		0.79	0.47	0.55	0.82
INQ-11*	3.12	2.23		**0.04**	0.46	0.04	0.82
INQ-12*	3.02	2.21		**−0.01**	0.42	–0.02	0.83
INQ-13r	4.11	2.26		0.78	0.38	0.49	0.83
INQ-14r	4.86	2.10		0.81	0.50	0.52	0.82
INQ-15r	4.30	2.28		0.78	0.40	0.47	0.83

Bold indicates unacceptable correlation value.

*We mentioned separate two structures, i.e., INQ 1-6 for perceived burdensomeness and INQ 7–15 for thwarted belongingness.

**FIGURE 2:**
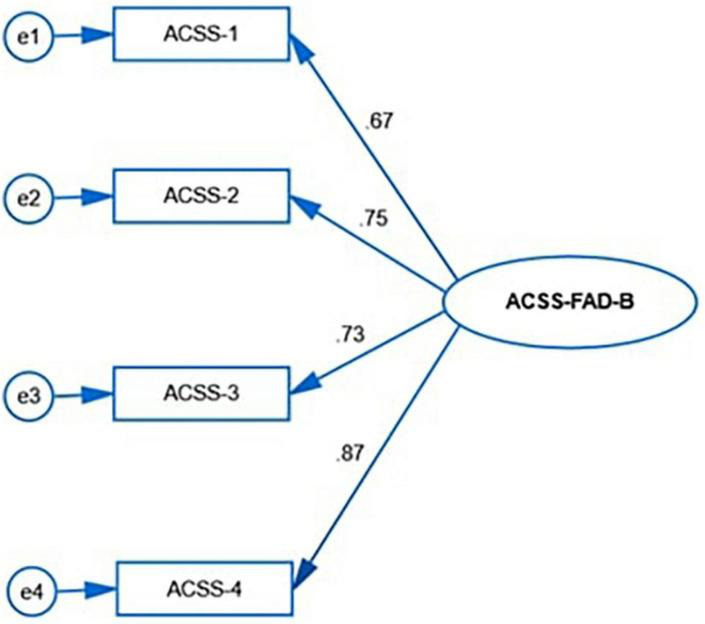
First order confirmatory factor analysis ACSS-FAD-B.

The mean score of responses of the items in ACSS-FAD-B is presented in Table 3. The factor rotation revealed acceptable values (0.36–0.89) in items except three items (item 1, 4, and 6) took poor loading (0.04, 0.05, and 0.18, respectively) (Table 3). These three items again showed poor inter-item correlation i.e., 0.04, 0.05, and 0.12 respectively (Table 3). Again, these three items revealed unacceptable corrected item-total correlation i.e., 0.24, 0.28, and 0.13. Based on these factors these three items have been dropped from the final construct. Therefore, the ACSS-FAD-B contains four items only as a single construct (Figure 2).

**TABLE 3:** Construct and factor loading of ACSS-FAD-B (*n* = 1,207).

Item	Mean	Std. deviation	Factor loading	Corrected item-total correlation	Inter-item correlation	Cronbach’s alpha if item deleted
ACSS-1*	0.98	1.51	0.04	0.24	**0.04**	0.65
ACSS-2r	1.62	1.68	0.87	0.44	1.00	0.59
ACSS-3r	1.75	1.67	0.89	0.52	0.70	0.56
ACSS-4*	1.32	1.60	0.05	0.28	**0.05**	0.64
ACSS-5r	1.94	1.67	0.82	0.46	0.57	0.58
ACSS-6*	2.36	1.67	–0.18	0.13	**−0.12**	0.68
ACSS-7	1.10	1.46	0.36	0.48	0.23	0.58

*Items dropped.

Bold indicates unacceptable correlation value.

We conducted the confirmatory factor analysis to assess the model fit of both INQ-B and ACSS-FAD-B which is presented in Table 4. Evaluation of the model fit for the absolute model of confirmatory factor analysis of INQ-B revealed χ^2^(53) = 258.31, *p* < 0.05 for INQ-B. The evidence of the absolute model resulted in an excellent fit. The evaluation for the current model fit indicated that the RMSEA and SRMR were 0.08 and 0.07, respectively, for INQ-B. On the other hand, the investigation of the null model comparison, i.e., GFI, CFI, and NFI were 0.94, 0.95, and 0.94, respectively; meanwhile, the value of the ratio of chi-square and degree of freedom (χ^2^/*df)* was found to be 4.87 for INQ-B The evaluation for the current model fit of ACSS-FAD-B indicated that the RMSEA and SRMR were 0.06 and 0.05, respectively. On the other hand, the investigation of the null model comparison, i.e., GFI, CFI, and NNFI were 0.99, 0.99, and 0.96, respectively; meanwhile, the value of the ratio of chi-square and degree of freedom (χ^2^/*df)* was found to be 4.15. Hence, the results suggested that the sample variance-covariance and population variance-covariance were consistent with the data for both of the scales.

**TABLE 4:** Fit indices of confirmatory factor analysis for INQ-B and ACSS-FAD-B (*n* = 1,207).

Scale	Model	χ^2^	*df*	χ^2^/*df*	*GFI*	*CFI*	*NFI*	*RMSEA*	*SRMR*	*P*-value
INQ-B	Model fit	258.31	53	4.87	0.94	95	0.94	0.08	0.07	<0.00001
ACSS-FAD-B	Model fit	8.31	2	4.15	0.99	99	0.98	0.06	0.05	0.0156

CFI, comparative fit index; NFI, normed fit index; RMSEA, root mean square error of approximation; SRMR, Standardized root mean square.

Perceived burdensomeness was significantly higher in female students (*p* = 0.001), among university students (*p* = 0.001), students with a history of mental illness (*p* = 0.001), family history of suicidal attempts (*p* = 0.001), family history of suicide (*p* = 0.003), and personal history of suicidal attempts (*p* = 0.001) (Table 5). Thwarted belongingness was significantly higher among students with past suicidal attempts (Table 5). Fearlessness about death was significantly higher among female students (*p* = 0.001), among non-Muslim participants (*p* = 0.001), and history of suicidal attempts (*p* = 0.001).

**TABLE 5:** Association between demography INQ-B and ACSS-FAD-B score.

		INQ Bangla		Perceived burdensomeness		Thwarted belongingness		ACSS-FAD	
					
Variable	n (%)	Median (IQR)	*P*-value	Median (IQR)	*P*-value	Median (IQR)	*P*-value	Median (IQR)	*P*-value
Total		38 (27–48)		7 (6–14)		27 (18–35)		6 (2–11)	
**Sex**									
Male	591 (49.09)	37 (27–47)	0.07	7 (6–12)	**<0.001**	26 (18–35)	0.97	5 (1–11)	**0.001**
Female	613 (50.91)	39 (27–48)		8 (6–15)		26 (18–35)		6 (3–12)	
**Education**									
HSC (Grade 12)	1,015 (84.30)	3 9 (28–48)	**0.01**	8 (6–14)	0.12	27 (18–35)	0.05	6 (2–12)	0.79
Graduate	189 (15.70)	35 (25–45)		7 (6–10)		25 (17–33)		6 (3–11)	
**Marital status**									
Unmarried	1,113 (92.44)	38 (2–48)	0.82	7 (6–14)	0.68	27 (18–35)	0.49	6 (1–12)	0.84
Married	91 (7.56)	38 (30–48)		6 (6–15)		26 (16–35)		6 (2–11)	
**Religion**									
Islam	1,052 (87.38)	38 (27–48)	0.56	7 (6–13)	0.25	27 (18–35)	0.17	6 (2–11)	**<0.001**
Others	152 (12.62)	36 (27–48)		8 (6–16)		26 (17–33)		8.5 (3.5–12)	
**Faculty**									
Medicine	485 (40.28)	38 (27–48)	0.49	6 (6–12)	**0.001**	27 (18–35)	0.74	6 (2–12)	0.24
Others	719 (59.72)	38 (28–48)		8 (6–15)		27 (18–35)		6 (2–11)	
**Family type**									
Nuclear	1,004 (83.39)	38 (27–48)	0.53	8 (6–14)	0.15	27 (18–35)	0.78	6 (2–12)	0.44
Joint	200 (16.61)	38.5 (28–45)		7 (6–12)		28 (18–34)		6 (2–10)	
**History of mental illness**									
Yes	139 (11.54)	44 (33–52)	**<0.001**	14 (7–24)	**<0.001**	27 (20–35)	0.51	7 (2–12)	0.16
No	1,065 (88.46)	37 (27–47)		7 (6–12)		27 (18–35)		6 (2–11)	
**Family history of suicide attempt**									
Yes	141 (11.71)	41 (31–50)	**0.003**	11 (6–21)	**<0.001**	27 (20–33)	0.48	6 (2–11)	0.91
No	1,063 (88.29)	38 (27–48)		7 (6–12)		27 (18–35)		6 (2–12)	
**Family history of suicide**									
Yes	66 (5.48)	38 (27–48)	**0.011**	10.5 (6–23)	**0.003**	29 (21–35)	0.30	6.5 (2–11)	0.85
No	1,138 (94.52)	41.5 (30–55)		7 (6–13)		27 (18–35)		6 (2–11)	
**History of suicide attempt**									
Yes	152 (12.62)	46 (33.5–55.5)	**<0.001**	15 (8–25)	**<0.001**	29 (20.5–36)	**0.025**	8 (3–12)	**<0.001**
Others	1,052 (87.38)	37 (26–47)		7 (6–12)		27 (18–34.5)		6 (2–11)	

*p*-value was determined by Mann-Whitney *U* test. Significant *p*-values are shown in bold.

## Discussion

### Main findings of the study

This study aimed to validate the INQ-15 and ACSS-FAD into Bangla along with the assessment of the level of perceived burdensomeness, thwarted belongingness, and fearlessness of death among university level students in Bangladesh. The study revealed acceptable levels of internal consistency of all the constructs i.e., INQ-B (α = 0.84), with sub-domains perceived burdensomeness (α = 0.92), thwarted belongingness (α = 0.87), and ACSS-FAD-B (α = 0.75). The exploratory factor analysis revealed an acceptable sample size for the validation study measured by KMO Measure of Sampling Adequacy (24). The final INQ-B contains two domains with 12 items, 6 items in each PB, and TB, and ACSS-FAD-B with a one-dimensional structure consisted of four items. Perceived burdensomeness was higher in females (*p* = 0.001), university students (*p* = 0.001), students with a history of mental illness (*p* = 0.001), a family history of suicidal attempts (*p* = 0.001), a family history of suicide (*p* = 0.003), and a personal history of suicidal attempt(s) (*p* = 0.001). Thwarted belongingness was higher among students with past suicidal attempts. Fearlessness about death was higher among females (*p* = 0.001), non-Muslim participants (*p* = 0.001), and a history of suicidal attempts (*p* = 0.001). We found a similar distribution of age, sex, marital status, and suicidal behavior in our previous study among the university students (22). The study conducted in India among 432 undergraduate students with the mean age 19.41 ± 1.54 years where the same three items (item 9, 11, and 12) were excluded from the TB domain of INQ-15 due to the poor factor loading (25). This may reflect the cultural variation of Western culture and South-Asian culture. These three items reflect the social disconnection which perhaps differently perceived in Western and South-Asian culture. However, these three items had also poor loading in German validation study that warrants further explorations (26). The reliability status of Bangla constructs revealed acceptable and similar findings like Indian constructs, PB (α = 0.90), TB (α = 0.84) (20), and German validation study (26). The Cronbach’s alpha of ACSS-FAD-7 revealed 0.69 when it was assessed among the 119 veterans (27).

The higher level of PB among the females could be explained by the patriarchal societal norms in Bangladesh where females have lower economic and social freedom. Additionally, it could be due to the personality constructs. However, further studies are warranted to ascertain the direction of the association. The lower level of PB among the medical students than the university students could be due to the academic environment as medical students are usual to consider human psychology, emotions, and behavior. A higher stigma and lower mental health literacy could be attributed to the higher level of PB among the students with a history of mental illness. One previous study assessing the relationship between psychiatric disorders and TB and PB revealed having a psychiatric disorder was significantly associated with PB and TB (28).

In the current study, we cannot explain the higher fearlessness among females and we recommend further studies to explore the finding. However, we speculate that based on religious background Muslims reveal lower fearlessness. Past suicide attempt was significantly associated with all the three components of IPTS in Bangladesh among students. Previous studies revealed that any previous attempt reduce the fear of further attempts which explain the higher fearlessness among respondents with past attempts (7, 29, 30).

### Implications of study findings

The study has several implications. Firstly, it validates the two vital instruments of suicidology in Bangla that opens newer avenues for further research in Bangladesh which in turn will bolster the research and reinforce the formulation of suicide prevention strategies of the country. Additionally, it revealed the level of thwarted belongingness, perceived burdensomeness, and fearlessness about death among university students of Bangladesh indicating an association between the status and past suicide attempts. However, we cannot assess the direction of the association due to the study design. Therefore, this could be explained in a bidirectional way. The suicidal attempts could be attributed by the higher level of thwarted belongingness, perceived burdensomeness, and fearlessness about death. In the other way round, the higher level of thwarted belongingness, perceived burdensomeness, and fearlessness about death could be due to the previous attempts. Further studies are warranted to explain the association as the presence of higher scores in all three domains indicates a precursor of high suicide risk (13).

### Strengths and limitations

This is the first study assessing the psychometric properties of the INQ-B and ACSS-FAD-B. Additionally, it also determined the association of thwarted belongingness, perceived burdensomeness, and acquired capability for suicide with different sociodemographic variables in Bangladesh. However, several important limitations should be considered. Firstly, we assessed only one form of reliability i.e., internal consistency without assessing the test-retest, and inter-rater forms of reliability. Secondly, we applied the instruments to a specific group of participants i.e., university students which may restrict the generalization of study findings. Thirdly, institutions were chosen purposively and responses were collected conveniently which may be a source of selection and response bias hindering the generalization of study findings. Fourthly, participants in the study were young, in their active studentship which may be different from participants living in a rural area, with different occupations and living statuses. Fifthly, we did not assess the convergent and divergent validities due to the unavailability of validated instruments in Bangla.

## Conclusion

This study found an acceptable reliability and validity of two suicide scales (INQ and ACSS-FAD) in Bangla that can be used in subsequent research projects. The final INQ-B pertained six items in each domain (total 12 items) and ACSS-FAD retained four items. Prevention strategies targeting to females, persons with psychiatric disorder, history of previous attempt(s) should be prioritized specially among the young age group. Further studies in different group of populations are warranted to assess the generalizability of the instruments.

## Data availability statement

The raw data supporting the conclusions of this article will be made available by the authors, without undue reservation.

## Ethics statement

This study was approved by the Ethical Review Committee of Enam Medical College on 07 March 2022 (EMC/ERC/2022/03-2). The patients/participants provided their written informed consent to participate in this study.

## Author contributions

SA contributed to the idea, concept, design, data analysis plan, and drafting of manuscript. FH contributed to the questionnaire preparation and data collection. MZ and TT contributed to the questionnaire preparation and drafting of manuscript. MKI, FS, AK, MRI, AR, and NG contributed to the data collection. AM and MK contributed to the data analysis. All authors contributed to the manuscript revision, read, and approved the submitted version.
